# Performance of *T*^2^-based PCA mix control chart with KDE control limit for monitoring variable and attribute characteristics

**DOI:** 10.1038/s41598-024-58052-4

**Published:** 2024-03-28

**Authors:** Muhammad Ahsan, Muhammad Mashuri, Dedy Dwi Prastyo, Muhammad Hisyam Lee

**Affiliations:** 1 Deparment of Statistics, Institut Teknologi Sepuluh Nopember, Surabaya, Indonesia; 2 Department of Mathematical Sciences, Universiti Teknologi Malaysia, Johor Bahru, Malaysia

**Keywords:** Hotelling’s *T*^2^, Kernel density estimation, Mixed quality characteristics, Outlier, PCA mix, Engineering, Mathematics and computing

## Abstract

In this work, the mixed multivariate *T*^2^ control chart’s detailed performance evaluation based on PCA mix is explored. The control limit of the proposed control chart is calculated using the kernel density approach. Through simulation studies, the proposed chart’s performance is assessed in terms of its capacity to identify outliers and process shifts. When 30% more outliers are included in the data, the proposed chart provides a consistent accuracy rate for identifying mixed outliers. For the balanced percentage of attribute qualities, misdetection happens because of the high false alarm rate. For unbalanced attribute qualities and excessive proportions, the masking effect is the key issue. The proposed chart shows the improved performance for the shift in identifying the shift in the process.

## Introduction

Statistical process control (SPC) is a statistical methodology for monitoring and controlling the variation of a process to ensure that it produces products that meet customer requirements. A control chart, which is part of SPC, is one of the tools often used to monitor the company’s quality of products and services^1^. Based on the number of monitored quality characteristics, the control charts are divided into two types: univariate and multivariate control charts. The univariate control charts monitor only one quality characteristic, while the multivariate control charts are applied to monitor more than one quality characteristic.

In the current industrial era 4.0, it is hoped that a process can not only be monitored from one type of quality characteristic. For example, in monitoring the variable characteristics (in a numerical scale such as height or weight), a control variable chart is used. Meanwhile, attribute control charts are always employed to monitor categorical or attribute data (such as color or hardness)^2^. Monitoring a mixed quality characteristic in the manufacturing process is important^3^. However, the monitoring procedure for mixed quality characteristics was commonly conducted in individual ways in the past. The inefficiency will happen due to the need for calculating two statistics and control limits. Consequently, the administrator will have hardship in determining the monitoring result if the two procedures yield a different result. Therefore, a new concept of monitoring mixed characteristics is urgently needed.

Ahsan et al.^4^ proposed a new monitoring procedure based on the PCA Mix algorithm to overcome this issue. This work also extended to detecting outliers for various numbers of contaminated outliers^5^. The *T*^2^ statistics are used to form the control chart in this method. Meanwhile, due to the unknown distribution, the control limit of the PCA Mix chart is estimated using the kernel density, a non-parametric method to estimate the empirical density from the unknown distribution^6^. However, in this work, the performance of the PCA Mix chart is only evaluated for one categorical data or attribute characteristic in detecting outliers. Additionally, both variable and attribute qualities are tracked in the effectiveness of the PCA Mix chart in identifying a change in the process. There is no suggestion for what shift this chart performs best, as a result.

Based on those reasons, this work is proposed to evaluate in detail the performance of the PCA Mix chart for detecting outliers and shift in the process. Similar to the PCA Mix chart proposed by Ahsan et al.^4^, the proposed chart also employed the kernel density estimation (KDE) in calculating the control limit. The proposed chart is evaluated for more than one attribute characteristic detecting outliers. On the other hand, the proposed chart is evaluated for a different kind of shift and correlation when the process change is being monitored. In this work, it is also shown how the proposed chart is used to monitor actual data and how its performance is compared.

The remaining portions of this work are structured as follows: Sect. “Related works” reports the connected works of this research. The charting processes for the suggested method were provided in Sect. “PCA mix”. In Sections “Charting procedures” and “Performance in detecting outlier”, performance assessments for identifying outliers and process adjustments are presented. Furthermore, Sect. “Performance evaluation in monitoring process shift” illustrates how the suggested strategy is used to track the actual dataset. Section “Application in the real cases” provides a summary of the conclusion.

## Related works

Recent advancements in the control chart are discussed in this section. This section covers three different categories of control charts: multivariate variable charts, attribute charts, and mixed charts. Three different multivariate control chart types such as Hotelling’s *T*^2^, Multivariate EWMA, and Multivariate CUSUM are the main emphasis of this development. The three different multivariate variable charts’ recent developments are summarized in Table 1. Table 2 lists the most current attribute chart works. The table demonstrates that current research has mostly concentrated on attribute charts using fuzzy, Poisson, and multinomial data. Recent advancements in the control chart are discussed in this section. In this section, the multivariate variable chart, attribute chart, and flow chart are the three primary forms of control charts that are covered.

**Table 1:** Multivariate variable chart’s most recent advancement.

References	Method	Highlight
Haq and Khoo^7^	New Adaptive MEWMA chart	The proposed chart can detect small and moderate shifts in the mean of a multivariate normal process
Ahmad and Ahmed^8^	*T*^*2*^ control chart for high-dimensional data	The suggested approach may be used with great accuracy without any preprocessing or dimension reduction
Yenageh et al.^9^	Adaptive MEWMA Approach for Monitoring Linear and Logistic Profiles	The proposed chart performs better in monitoring Linear and Logistic Profiles
Haddad^10^	Mahalanobis distance-modified *T*^*2*^ control charts	Comparing the suggested approach to the standard chart, it has an advantage in recognizing more outliers
Maleki et al.^11^	*T*^*2*^ control chart with robust estimators for the median	When compared to the traditional chart, the proposed technique performs better
Mashuri et al.^12^	*Tr* (*R*^2^) control charts	For large features and sample sizes, the suggested control chart technique performs better at detecting shifts
Mehmood et al.^13^	Hotelling *T*^2^ control chart based on bivariate ranked set	When compared to the standard Hotelling *T*^2^ scheme, the proposed control chart approaches perform remarkably well
Tran and Khoo^14^	MEWMA-CoDa chart	Measurement mistakes can be handled using the proposed control chart approach to find process changes
Haq and Khoo^15^	Adaptive MEWMA chart	The suggested chart outperforms the current adaptive multivariate charts in terms of performance
Haq et al.^16^	Dual MCUSUM with auxiliary	In comparison to the DMCUSUM and MDMCUSUM charts, the suggested chart performs better when identifying shifts of various magnitude in the process mean vector
Khusna et al.^17^	Residual-based Max MCUSUM	The method results in a more sensitive detection of mean compared to variance
Haq^18^	Weighted adaptive MCUSUM charts	In identifying a change in mean, proposed charts outperform the traditional MCUSUM
Leoni et al.^19^	*T*^*2*^ control chart for autocorrelated data	Bivariate *T*^2^ control chart for autocorrelated data

**Table 2:** Attribute chart’s most recent advancement.

References	Method	Highlight
Yeganeh et al.^20^	Run rules and MEWMA	For modest and moderate shifts in monitoring linear profiles, the proposed technique performs better
Xie et al.^21^	MCUSUM	For the majority of shift domains, the suggested chart performs better than the others
Mashuri et al.^22^	Fuzzy bivariate chart	Compared to the traditional bivariate Poisson chart, the suggested chart is more sensitive
Zhou et al.^23^	Synthetic chart	The suggested chart shows improved detection performance for both modest and big mean changes
Quinino et al.^24^	Attribute chart for monitoring of mean and variance	Comparing the suggested method to the conventional approach, the new way is simpler to implement
Aldosari et al.^25^	Multiple dependent state repetitive sampling (MDSRS)	The suggested technique performs better than the traditional strategy based on repetitive sampling
Aslam et al.^26^	Shewhart neutrosophic attributes chart	The suggested attribute control chart is effective at identifying changes in the process
Chong et al.^27^	Multi-attribute CUSUM-np chart	The proposed method performs as well as or better than the traditional chart
Aslam^28^	Attribute chart with the repetitive sampling using the neutrosophic approach	Compared to the current chart, the suggested chart with recurrent sampling under the neutrosophic system is better capable of detecting a change in the process
Wibawati et al.^29^	Fuzzy multinomial (FM) chart	FM chart is capable of detecting shifts
Ahsan et al.^30^	Laney *p*’ chart	The proposed *p*’ chart has a better performance for the moderate sample size and large for a different number of subgroups
Lee et al.^31^	Multinomial generalized likelihood ratio (MGLR) chart	The suggested chart performs better than the collection of 2-sided Bernoulli CUSUM charts
Aslam et al.^32^	Attribute control chart using multiple dependent state sampling	Compared to the traditional np chart, the proposed technique performs better

Additionally, Table 3 displays the mixed control chart’s most recent evolution. It is clear that a few works have looked at the mixed monitoring variable and attribute features in this field. Consequently, additional advancement in this field is required. In order to improve the monitoring process technique, this research aims to build and evaluate the performance of the mixed type chart, particularly the PCA mix control chart.

**Table 3:** Mixed chart’s most recent advancement.

References	Method	Highlight
Ahsan et al.^5^	PCA Mix chart	Comparing the proposed chart to other robust and traditional charts, it performs excellently in detecting more outliers with a larger percentage of outliers included
Ahsan et al.^4^	PCA Mix chart	When a suitable number of primary components are chosen, the suggested chart displays strong performance
Wang Su et al.^33^	Multivariate sign chart	Simulations demonstrate how effective the suggested control chart is in inspecting mixed-type data
Aslam Azam et al.^34^	Mixed chart	The mixed chart displays good monitoring process performance

## PCA mix

A statistical method called multivariate data analysis can be used to examine data that includes two or more quality factors. These qualities may either be attribute- or attribute-variable (interval- or ratio-based) (category). A statistical technique known as principal component analysis (PCA) is used to reduce the dimensions of continuous data, also known as variable characteristics in statistical process control (SPC). An extension of correspondence analysis (CA), multiple correspondence analysis (MCA) examines the relationships between a number of correlated categorical variables, also known as attribute characteristics in SPC. When the observations are categorical, MCA may be thought of as an extension of the PCA approach^35^. Thus, PCA Mix method is a combination of PCA and MCA that can be used to handle different types of quality characteristics together.

In this study, the PCA Mix technique is implemented in accordance with the strategy suggested by Chavent et al.^36^. Let $n \times p$ matrix ${\mathbf{X}}_{1}$ and $n \times q$ matrix ${\mathbf{X}}_{2}$ consist of variable and attribute characteristics, respectively, where *n* is the number of observations, *p* is the number of variable characteristics, and *q* is the number of attribute characteristics. An indicator matrix ${\mathbf{G}}$ with dimensions $n \times m$ provides binary coding for each attribute’s degree of features, where *m* is the sum of all attribute level features. An $n \times (p + m)$ matrix ${\mathbf{Z}} = [{\mathbf{Z}}_{1} ,{\mathbf{Z}}_{2} ]$ includes a real number component, where ${\mathbf{Z}}_{1}$ and ${\mathbf{Z}}_{2}$ are centred matrices of ${\mathbf{X}}_{1}$ and ${\mathbf{G}}$. ${\tilde{\mathbf{Z}}}$ is calculated as

1 $$ {\mathbf{\tilde{Z} = N}}^{{\frac{{\mathbf{1}}}{{\mathbf{2}}}}} {\mathbf{ZM}}^{{\frac{{\mathbf{1}}}{{\mathbf{2}}}}} , $$

where ${\mathbf{\rm N}} = \frac{1}{n}{\mathbf{I}}_{n}$ is the rows’ weights of **Z**, ${\mathbf{M}} = diag\left( {1,...,1,\frac{n}{{n_{1} }},...,\frac{n}{{n_{m} }}} \right)$ is the weights of the columns of **Z**, the first *p* columns of **Z** are weighted by 1, and the last *m* columns are weighted by $\frac{n}{{n_{s} }},$ for $s = 1,2, \ldots ,m.$ The next step is solving the eigenvalue problem of ${\tilde{\mathbf{Z}}}$ using the Generalized Singular Value Decomposition (GSVD) in Chavent et al.^36^ as

2 $$ {\tilde{\mathbf{Z}}} = {\mathbf{U\Lambda V}}^{T} , $$

where ${{\varvec{\Lambda}}} = {\text{diag}}(\sqrt {\lambda_{1} } ,\sqrt {\lambda_{2} } , \ldots \sqrt {\lambda_{r} } ),$ where $\lambda_{1} ,\lambda_{2} , \ldots ,\lambda_{r}$ are the eigenvalues of ${\tilde{\mathbf{Z}}},$ and *r* denotes the rank of ${\tilde{\mathbf{Z}}}.$ Matrix ${\mathbf{U}}$, which has $n \times r$ dimensions, is an eigenvector of ${\tilde{\mathbf{Z}}}$, and ${\mathbf{V}}$ is the $(p + m) \times r$ matrix of the eigenvectors of ${\tilde{\mathbf{Z}}}.$ As a result, the principal component of PCA mix may be calculated as

3 $$ {\mathbf{Y}}^{mix} = \,{\mathbf{ZMV}}. $$

with the size of $n \times r.$

## Charting procedures

**Figure 1:**
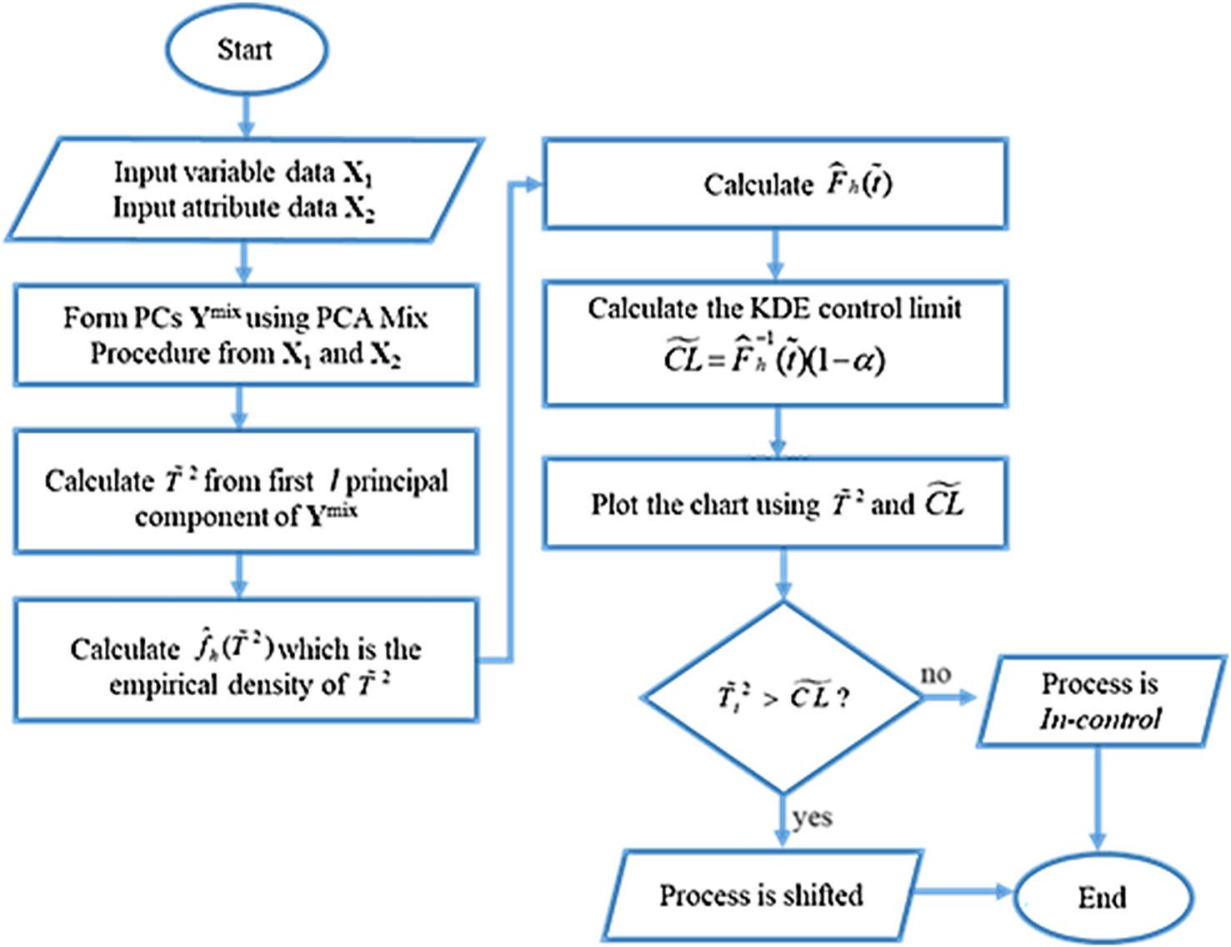
PCA mix control chart procedures.

The steps to create a multivariate control chart based on PCA Mix are covered in this section. The steps for building a multivariate control chart based on PCA Mix are shown in Fig. 1. There are three basic phases in the process. The PCs are initially calculated from the combined features using PCA Mix. The *T*^2^ statistics are computed in the second phase using certain main components. Finally, use KDE to estimate the suggested chart’s control limit.

**Table:** 

PCA mix control chart’s procedures
**Step 1** Input the variable data **X**_1_ and the attribute data **X**_2_
**Step 2** Calculate the principal component scores (PCs) mix, denoted as ${\mathbf{Y}}^{mix}$, using the PCA Mix method from **X**_1_ and **X**_2_
**Step 3** Take the first *v* components and calculate $\tilde{T}_{i}^{2} = \sum\limits_{v = 1}^{l} {\frac{{(y_{i,v}^{mix} - \tilde{\mu }_{v} )}}{{\lambda_{mix,v}^{{}} }}^{2} } ,$ where $\lambda_{v}$ is the eigenvalue for the v-th PCs
**Step 4** Calculate the empirical density of $\tilde{T}_{i}^{2}$ statistics, $\hat{f}_{h} (\tilde{T}_{{}}^{2} ) = \frac{1}{{n\widehat{h}}}\sum\limits_{i = 1}^{n} {k\left( {\frac{{T_{{}}^{2} - \tilde{T}_{i}^{2} }}{{\widehat{h}}}} \right)}$, where $\widehat{h}$ is the optimum bandwidth calculated using Botev, Grotowski, and Kroese algorithm ^37^
**Step 5** Calculate the distribution function $\tilde{T}_{i}^{2}$ statistics, $\widehat{F}_{h}^{{}} (\widetilde{t}) = \int\limits_{0}^{{\widetilde{t}_{{}}^{2} }} {\hat{f}_{h} (\tilde{T}_{{}}^{2} )d} \tilde{T}_{{}}^{2}$
**Step 6** Calculate the KDE control limit $\widetilde{CL} = \widehat{F}_{h}^{ - 1} (\widetilde{t})(1 - \alpha )$, when process is in-control
**Step 7** Plot the $\tilde{T}_{i}^{2}$ along with KDE control limit $\widetilde{CL}$ to form the PCA Mix Control Chart

## Performance in detecting outlier

The effectiveness of the proposed chart in identifying outliers mingled with the in-control data is demonstrated in this section. Simulated studies involving various situations are carried out to evaluate its performance. For the simulations, the variable characteristics are assumed to follow the multivariate normal distribution ${\mathbf{X}}_{1} \sim N_{p} ({\mathbf{0,I}})$, while the attribute characteristics are generated to follow the multinomial distribution with three categories ${\mathbf{X}}_{2} \sim M(\theta_{1} ,\theta_{2} ,\theta_{3} )$. Similar to Ahsan et al.^5^, the attribute characteristics are differentiated into three types such as the almost balanced proportion ($\theta_{1} ,\theta_{2} = 0.3{\text{ and }}\theta_{3} = 0.4$), the imbalanced proportion ($\theta_{1} ,\theta_{2} = 0.1{\text{ and }}\theta_{3} = 0.8$), and the extreme imbalanced proportion ($\theta_{1} ,\theta_{2} = 0.05{\text{ and }}\theta_{3} = 0.9$).

For the detailed performance, the number of attribute characteristics is evaluated for 2, 3, and 5. On the other hand, 5 variable characteristics is used with the number of observations *n* = 1000. The outliers mixed with the clean data are set to 5, 10, 20, 30, 40, and 50 percent out of the total observations. The proposed chart’s accuracy may be assessed using the confusion matrix by categorizing the findings into four groups: true positives (TP), true negatives (TN), false positives (FP), and false negatives (FN) (FN). The examples that were successfully identified as outliers are denoted by the letters TP, TN, FP, and FN, whereas the instances that were wrongly identified as outliers and not outliers are denoted by the letters FN and FP. The hit rate (HR), which can be computed using Eq. (4), is the accuracy level employed.

4 $$ {\text{HR}} = \frac{{{\text{TP}} + {\text{TN}}}}{{{\text{TP}} + {\text{TN}} + {\text{FP}} + {\text{FN}}}}. $$

False positive rate (FPR) and false negative rate (FNR) are two categories under which the mistake rate in a confusion matrix may be subdivided. The percentage of cases that are wrongly labeled as positive is known as the FPR, whereas the percentage of instances that are incorrectly classed as negative is known as the FNR. Equations (5) and (6), respectively, are used to determine the FPR and FNR formulas:

5 $$ {\text{FNR}} = \frac{{{\text{FN}}}}{{{\text{TP}} + {\text{FN}}}}, $$

6 $$ {\text{FPR}} = \frac{{{\text{FP}}}}{{{\text{TN}} + {\text{FP}}}}. $$

The detailed algorithm for simulation studies can be found in Ahsan et al.^5^.

### Two attribute characteristics

Table 4 shows the performance of the proposed chart in detecting outliers for two attribute characteristics with $\theta_{1} ,\theta_{2} = 0.3{\text{ and }}\theta_{3} = 0.4.$ In general, the proposed chart still has a stable performance for no more than 30 percent outlier added to the clean data. For this case, it can be seen that the misdetection occurs due to a large number of the in-control data declared as an outlier (high FP rate). The proposed chart performance in detecting outliers for two attribute characteristics with imbalanced proportion is reported in Table 5. Unlike the previous case (two variables with balanced proportion), the misdetections are caused by the inability of the control chart to capture the actual outliers, which can be seen from the high FN rate. Furthermore, Table 6 presents the performance of the proposed chart to detect outliers for the extreme imbalanced proportion ($\theta_{1} ,\theta_{2} = 0.05{\text{ dan }}\theta_{3} = 0.9$). For this condition, it can be seen that the high value of the FN rate causes a low level of accuracy in the proposed chart. In general, using the number of components *l* = 2 produces better results for this case.

**Tabel 4:** Performance of the proposed chart in identifying outliers for two attribute characteristics with $\theta_{1} ,\theta_{2} = 0.3{\text{ and }}\theta_{3} = 0.4$.

Number of components *l*	Outlier 5%			Outlier 10%		
	HR	FNR	FPR	HR	FNR	FPR
*l* = 2	0.96966	0.0000	0.0319	0.95122	0.0002	0.0542
*l* = 3	0.97353	0.0000	0.0279	0.95445	0.0005	0.0506
*l* = 4	0.96633	0.0000	0.0355	0.94993	0.0001	0.0556

	Outlier 20%			Outlier 30%		
	HR	FNR	FPR	HR	FNR	FPR
*l* = 2	0.86096	0.0020	0.1733	0.78931	0.0216	0.2917
*l* = 3	0.90388	0.0033	0.1193	0.78449	0.0204	0.2991
*l* = 4	0.88373	0.0034	0.1445	0.79002	0.0230	0.2901

	Outlier 40%			Outlier 50%		
	HR	FNR	FPR	HR	FNR	FPR
*l* = 2	0.64591	0.0893	0.5306	0.49819	0.2534	0.7502
*l* = 3	0.64703	0.0860	0.5310	0.50135	0.2661	0.7312
*l* = 4	0.65355	0.0939	0.5148	0.50137	0.2373	0.7600

**Table 5:** Performance of the proposed chart in identifying outliers for two attribute characteristics with $\theta_{1} ,\theta_{2} = 0.1{\text{ and }}\theta_{3} = 0.8$

Number of components *l*	Outlier 5%			Outlier 10%		
	HR	FNR	FPR	HR	FNR	FPR
*l* = 2	0.99487	0.0132	0.0047	0.99012	0.0260	0.0081
*l* = 3	0.98997	0.1972	0.0002	0.96837	0.3140	0.0003
*l* = 4	0.97897	0.2988	0.0064	0.95275	0.4135	0.0066

	Outlier 20%			Outlier 30%		
	HR	FNR	FPR	HR	FNR	FPR
*l* = 2	0.96786	0.0992	0.0154	0.88818	0.2901	0.0354
*l* = 3	0.89210	0.5359	0.0009	0.76512	0.7760	0.0030
*l* = 4	0.86946	0.6205	0.0080	0.74975	0.8068	0.0117

	Outlier 40%			Outlier 50%		
	HR	FNR	FPR	HR	FNR	FPR
*l* = 2	0.72744	0.5326	0.0992	0.50086	0.7921	0.2062
*l* = 3	0.63372	0.8995	0.0108	0.50003	0.9693	0.0307
*l* = 4	0.62190	0.9167	0.0190	0.50082	0.9614	0.0370

**Table 6:** Performance of the proposed chart in identifying outliers for two attribute characteristics with $\theta_{1} ,\theta_{2} = 0.05{\text{ and }}\theta_{3} = 0.9$

Number of components *l*	Outlier 5%			Outlier 10%		
	HR	FNR	FPR	HR	FNR	FPR
*l* = 2	0.97936	0.3600	0.0028	0.94568	0.5218	0.0024
*l* = 3	0.95731	0.8080	0.0024	0.91529	0.8210	0.0029
*l* = 4	0.95375	0.8480	0.0041	0.90554	0.9110	0.0037

	Outlier 20%			Outlier 30%		
	HR	FNR	FPR	HR	FNR	FPR
*l* = 2	0.84812	0.7440	0.0039	0.72375	0.9128	0.0034
*l* = 3	0.81917	0.8886	0.0039	0.70786	0.9673	0.0028
*l* = 4	0.81048	0.9301	0.0044	0.70713	0.9621	0.0060

	Outlier 40%			Outlier 50%		
	HR	FNR	FPR	HR	FNR	FPR
*l* = 2	0.60831	0.9717	0.0050	0.50009	0.9920	0.0078
*l* = 3	0.60242	0.9892	0.0032	0.50004	0.9889	0.0111
*l* = 4	0.60167	0.9875	0.0055	0.49978	0.9917	0.0087

### Three attribute characteristics

Proposed chart performance in outlier detection for three balanced attribute characteristics $\theta_{1} ,\theta_{2} = 0.3{\text{ and }}\theta_{3} = 0,4$ is presented in Table 7. Similar to the two attribute characteristics case, for this case, the misdetection happens due to the high false alarm produced represented by the high value of FP rate. Tables 8 and 9 show the performance for three attribute characteristics with imbalanced and extreme imbalanced proportions, respectively. In this case, it can be seen that the misdetection for these two cases happens due to the actual outliers are failed to be detected, represented by the high value of the FN rate. From this case, it also can be seen that using smaller principal components produces better results. The performance degradation can be seen when the proposed chart monitors more than 30 percent of outliers. Also, the more imbalanced proportion of the attribute characteristics, the higher the accuracy level produced.

**Table 7:** Performance of the proposed chart in identifying outliers for three attribute characteristics with $\theta_{1} ,\theta_{2} = 0.3{\text{ and }}\theta_{3} = 0.4$.

Number of components *l*	Outlier 5%			Outlier 10%		
	HR	FNR	FPR	HR	FNR	FPR
*l* = 2	0.96961	0.0004	0.0320	0.95411	0.0001	0.0510
*l* = 3	0.94098	0.0000	0.0621	0.92895	0.0003	0.0789
*l* = 4	0.94272	0.0000	0.0603	0.91451	0.0000	0.0950

	Outlier 20%			Outlier 30%		
	HR	FNR	FPR	HR	FNR	FPR
*l* = 2	0.90321	0.0030	0.1202	0.77372	0.0193	0.3150
*l* = 3	0.81657	0.0005	0.2292	0.71108	0.0106	0.4082
*l* = 4	0.81361	0.0010	0.2327	0.70425	0.0111	0.4177

	Outlier 40%			Outlier 50%		
	HR	FNR	FPR	HR	FNR	FPR
*l* = 2	0.62677	0.0711	0.5746	0.49915	0.2422	0.7595
*l* = 3	0.60148	0.0522	0.6294	0.50158	0.1654	0.8314
*l* = 4	0.60654	0.0587	0.6167	0.49921	0.2002	0.8014

**Table 8:** Performance of the proposed chart in identifying outliers for three attribute characteristics with $\theta_{1} ,\theta_{2} = 0.1{\text{ and }}\theta_{3} = 0.8$.

Number of components *l*	Outlier 5%			Outlier 10%		
	HR	FNR	FPR	HR	FNR	FPR
*l* = 2	0.99409	0.0256	0.0049	0.98687	0.0625	0.0076
*l* = 3	0.85182	0.0548	0.1531	0.84175	0.1190	0.1626
*l* = 4	0.96442	0.2608	0.0237	0.93301	0.3925	0.0308

	Outlier 20%			Outlier 30%		
	HR	FNR	FPR	HR	FNR	FPR
*l* = 2	0.94115	0.1742	0.0300	0.83769	0.3934	0.0633
*l* = 3	0.80244	0.2704	0.1793	0.72151	0.4766	0.1936
*l* = 4	0.85371	0.5784	0.0383	0.74692	0.7264	0.0502

	Outlier 40%			Outlier 50%		
	HR	FNR	FPR	HR	FNR	FPR
*l* = 2	0.67938	0.6334	0.1121	0.50034	0.7424	0.2569
*l* = 3	0.60897	0.6680	0.2064	0.49729	0.7581	0.2473
*l* = 4	0.62444	0.7973	0.0944	0.49841	0.8594	0.1438

**Tabel 9:** Performance of the proposed chart in identifying outliers for three attribute characteristics with $\theta_{1} ,\theta_{2} = 0.05{\text{ and }}\theta_{3} = 0.9$.

Number of components *l*	Outlier 5%			Outlier 10%		
	HR	FNR	FPR	HR	FNR	FPR
*l* = 2	0.96109	0.3710	0.0214	0.9189	0.5667	0.0271
*l* = 3	0.88371	0.4712	0.0976	0.84522	0.6543	0.0993
*l* = 4	0.93443	0.7830	0.0278	0.88509	0.8596	0.0322

	Outlier 20%			Outlier 30%		
	HR	FNR	FPR	HR	FNR	FPR
*l* = 2	0.81906	0.7567	0.0370	0.70547	0.8617	0.0514
*l* = 3	0.76344	0.7958	0.0968	0.66809	0.8776	0.0981
*l* = 4	0.79227	0.8775	0.0403	0.68634	0.8911	0.0662

	Outlier 40%			Outlier 50%		
	HR	FNR	FPR	HR	FNR	FPR
*l* = 2	0.60186	0.9019	0.0623	0.70547	0.8617	0.0514
*l* = 3	0.58202	0.8975	0.0983	0.49999	0.9005	0.0995
*l* = 4	0.59934	0.9148	0.0579	0.50044	0.9363	0.0628

### Five attribute characteristics

Table 10 shows the outlier monitoring results for five attribute data with $\theta_{1} ,\theta_{2} = 0.3{\text{ and }}\theta_{3} = 0.4.$ According to the simulation results, it can be concluded that, in this case, the misdetection occurs due to a large number of the in-control data declared as an outlier (see FP rate). The performances of the proposed chart for $\theta_{1} ,\theta_{2} = 0.1{\text{ and }}\theta_{3} = 0.8$ as well as $\theta_{1} ,\theta_{2} = 0.05{\text{ and }}\theta_{3} = 0.9$ are reported in Tables 11 and 12, respectively. Similar to the two previous cases, the failure to detect the actual outliers leads to reduced accuracy given by the proposed chart. In general, the usage of the smaller principal component leads to higher accuracy. This chart is still at its peak performance for less than 40 percent outlier mixed. Moreover, the more imbalanced proportion of the attribute characteristics monitored by the proposed chart, the higher the Hit rate or accuracy produced.

**Table 10:** Performance of the proposed chart in identifying outliers for five attribute characteristics with $\theta_{1} ,\theta_{2} = 0.3{\text{ and }}\theta_{3} = 0.4$.

Number of components *l*	Outlier 5%			Outlier 10%		
	HR	FNR	FPR	HR	FNR	FPR
*l* = 2	0.99097	0.0010	0.0095	0.98861	0.0035	0.0123
*l* = 3	0.98939	0.0024	0.0110	0.98264	0.0040	0.0188
*l* = 4	0.98968	0.0016	0.0108	0.98590	0.0051	0.0151

	Outlier 20%			Outlier 30%		
	HR	FNR	FPR	HR	FNR	FPR
*l* = 2	0.96411	0.0226	0.0394	0.89619	0.0991	0.1058
*l* = 3	0.95652	0.0252	0.0480	0.87571	0.0956	0.1366
*l* = 4	0.95204	0.0249	0.0537	0.87924	0.1079	0.1263

	Outlier 40%			Outlier 50%		
	HR	FNR	FPR	HR	FNR	FPR
*l* = 2	0.73821	0.2665	0.2587	0.50111	0.5183	0.4794
*l* = 3	0.72134	0.2432	0.3023	0.49897	0.5168	0.4852
*l* = 4	0.72961	0.2916	0.2562	0.49995	0.5059	0.4942

**Table11:** Performance of the proposed chart in identifying outliers for five attribute characteristics with $\theta_{1} ,\theta_{2} = 0.1{\text{ and }}\theta_{3} = 0.8$.

Number of components *l*	Outlier 5%			Outlier 10%		
	HR	FNR	FPR	HR	FNR	FPR
*l* = 2	0.99532	0.0734	0.0011	0.98895	0.0938	0.0019
*l* = 3	0.99452	0.0712	0.0020	0.98482	0.1222	0.0033
*l* = 4	0.99122	0.1298	0.0024	0.97739	0.2033	0.0025

	Outlier 20%			Outlier 30%		
	HR	FNR	FPR	HR	FNR	FPR
*l* = 2	0.93924	0.2892	0.0037	0.82966	0.5474	0.0088
*l* = 3	0.93054	0.3266	0.0052	0.81657	0.5862	0.0108
*l* = 4	0.91527	0.4056	0.0045	0.79806	0.6460	0.0116

	Outlier 40%			Outlier 50%		
	HR	FNR	FPR	HR	FNR	FPR
*l* = 2	0.67401	0.7734	0.0277	0.49912	0.8989	0.1029
*l* = 3	0.66005	0.8123	0.0251	0.49972	0.9552	0.0453
*l* = 4	0.65823	0.8083	0.0308	0.50067	0.9425	0.0562

**Table 12:** Performance of the proposed chart in identifying outliers for five attribute characteristics with $\theta_{1} ,\theta_{2} = 0.05{\text{ and }}\theta_{3} = 0.9$.

Number of components *l*	Outlier 5%			Outlier 10%		
	HR	FNR	FPR	HR	FNR	FPR
*l* = 2	0.97440	0.4946	0.0009	0.93969	0.5961	0.0008
*l* = 3	0.96295	0.7232	0.0009	0.92563	0.7264	0.0019
*l* = 4	0.96174	0.7256	0.0021	0.91941	0.7836	0.0025

	Outlier 20%			Outlier 30%		
	HR	FNR	FPR	HR	FNR	FPR
*l* = 2	0.85488	0.7148	0.0027	0.7262	0.9055	0.0031
*l* = 3	0.82801	0.8496	0.0026	0.71267	0.9501	0.0033
*l* = 4	0.81226	0.9315	0.0018	0.70802	0.9659	0.0031

	Outlier 40%			Outlier 50%		
	HR	FNR	FPR	HR	FNR	FPR
*l* = 2	0.60786	0.9739	0.0043	0.49957	0.9945	0.0063
*l* = 3	0.60587	0.9762	0.0061	0.49957	0.9936	0.0073
*l* = 4	0.60291	0.9864	0.0042	0.50055	0.9915	0.0074

Based on the simulation results about the performance of the proposed chart in detecting outliers, the following findings can be written as follows:

1. In general, the proposed chart only has good capabilities when used to monitor data with 30 percent outliers.
2. When used to monitor attribute characteristics with balanced proportions, the chart’s performance decreases due to high false alarms or swamping effects.
3. When used to monitor attribute characteristics with imbalanced and extreme imbalanced, the proportion of diagram performance decreases due to high false negatives or masking effects.
4. The proposed chart is suitable for monitoring outliers in attribute data with imbalanced and extreme imbalance proportions.

## Performance evaluation in monitoring process shift

This part evaluates the proposed chart’s effectiveness in order to inspect the process shift. Similar to the preceding part, attribute characteristics are created using a multinomial distribution with three different types of proportions, and variable characteristics are generated using a multivariate normal distribution. In this instance, the performance of the suggested chart is assessed for several types of shifts, such as a change in either variable characteristics, an attribute characteristics shift, or a shift in both variable and attribute characteristics. A new kind of correlation is tested to see how well the suggested chart performs. Using the same approach as Ahsan et al.^4^, the ARL_1_ is estimated by shifting the variable characteristics by ${{\varvec{\upmu}}}_{shift} = {{\varvec{\upmu}}} + {{\varvec{\updelta}}}_{\mu } ,$ where ${{\varvec{\updelta}}}_{\mu } = {\mathbf{0}}{\mathbf{.1}}$ and shifting the attribute characteristics by ${{\varvec{\uptheta}}}_{shift} = [\theta_{1} - \delta_{\theta } ;\;\theta_{2} - \delta_{\theta } ;\;\theta_{3} + 2\delta_{\theta } ],$ where $\,\delta_{\theta } = 0.0025$.

### Shift in variable characteristics

The proposed chart’s performance is shown in Tables 13, 14 and 15 for the balanced, imbalanced, and extremely imbalanced proportions of attribute data, respectively. In general, using the KDE control limit, the proposed chart produces ARL_0_ at around 370 for the false alarm rate $\alpha = 0.00273$. For the shift in only variable characteristics, the proposed chart can capture the change in the process by producing the lower ARL_1_ for the larger shift given. For this case, better performance is achieved when it is used to monitor the balanced parameter of the attribute characteristics.

**Table 13:** ARLs for $\theta_{1} ,\theta_{2} = 0.3{\text{ and }}\theta_{3} = 0.4$ with shift in the variable characteristics for *p* = 5.

Shift		Number of components *l*		
${\delta }_{\mu }$	${\delta }_{\theta }$	*l* = 2	*l* = 3	*l* = 4
0.0	0.0000	**354.586**	**386.497**	**377.743**
0.1	0.0000	123.905	166.972	173.247
0.2	0.0000	74.363	93.708	97.509
0.3	0.0000	53.117	62.026	64.519
0.4	0.0000	41.313	44.420	46.182
0.5	0.0000	33.801	33.216	34.512
0.6	0.0000	28.601	25.460	26.433
0.7	0.0000	24.788	19.772	20.509
0.8	0.0000	21.872	15.422	15.979
0.9	0.0000	19.569	11.988	12.402
1.0	0.0000	17.706	9.209	9.506
1.1	0.0000	16.166	6.912	7.115
1.2	0.0000	14.873	4.983	5.105
1.3	0.0000	13.771	3.340	3.394
1.4	0.0000	12.821	1.923	1.918
1.5	0.0000	12.394	1.286	1.255

ARL_0_ is in bold.

**Table 14:** ARLs for $\theta_{1} ,\theta_{2} = 0.1{\text{ and }}\theta_{3} = 0.8$ with shift in the variable characteristics for *p* = 5.

Shift		Number of components *l*		
${\delta }_{\mu }$	${\delta }_{\theta }$	*l* = 2	*l* = 3	*l* = 4
0.0	0.0000	**353.337**	**357.652**	**381.162**
0.1	0.0000	275.777	139.082	153.414
0.2	0.0000	161.416	79.543	88.427
0.3	0.0000	111.341	52.900	59.276
0.4	0.0000	83.488	38.034	42.994
0.5	0.0000	65.763	28.574	32.631
0.6	0.0000	53.492	22.024	25.457
0.7	0.0000	44.493	17.221	20.196
0.8	0.0000	37.611	13.548	16.173
0.9	0.0000	32.179	10.648	12.997
1.0	0.0000	27.781	8.300	10.426
1.1	0.0000	24.148	6.361	8.302
1.2	0.0000	21.096	4.732	6.518
1.3	0.0000	18.496	3.345	4.998
1.4	0.0000	16.255	2.149	3.688
1.5	0.0000	15.246	1.610	3.098

ARL_0_ is in bold.

**Table 15:** ARLs for $\theta_{1} ,\theta_{2} = 0.05{\text{ and }}\theta_{3} = 0.9$ with shift in the variable characteristics for *p* = 5.

Shift		Number of components *l*		
${\delta }_{\mu }$	${\delta }_{\theta }$	*l* = 2	*l* = 3	*l* = 4
0.0	0.0000	**354.586**	**386.497**	**377.743**
0.1	0.0000	296.688	166.400	136.717
0.2	0.0000	175.388	97.125	79.222
0.3	0.0000	121.396	65.578	52.853
0.4	0.0000	91.309	47.898	38.002
0.5	0.0000	72.162	36.643	28.547
0.6	0.0000	58.906	28.852	22.002
0.7	0.0000	49.185	23.139	17.201
0.8	0.0000	41.752	18.769	13.531
0.9	0.0000	35.883	15.320	10.633
1.0	0.0000	31.132	12.528	8.287
1.1	0.0000	27.208	10.221	6.349
1.2	0.0000	23.911	8.283	4.721
1.3	0.0000	21.103	6.633	3.334
1.4	0.0000	18.682	5.210	2.139
1.5	0.0000	17.593	4.569	1.601

ARL_0_ is in bold.

### Shift in attribute characteristics

The performances of the proposed chart with the shift in the attribute characteristics for balanced, imbalanced, and extreme imbalanced proportion parameters are sequentially presented in Tables 16, 17 and 18. For this case, using the KDE control limit, it can be found that the performance of the proposed chart for the in-control state is stable (see the ARL_0_ value at around 370 for all scenarios $\alpha = 0.00273$). Although the proposed chart can capture process shifts that occur in the attribute characteristics, the ARL_1_ obtained does not drop as sharply as when detecting a shift in the variable characteristics. Also, the proposed chart performs better than existing chart, particularly when dealing with highly imbalanced data.

**Table 16:** ARLs for $\theta_{1} ,\theta_{2} = 0.3{\text{ and }}\theta_{3} = 0.4$ with shift in the attribute characteristics for *p* = 5.

Shift		Number of components *l*		
${\delta }_{\mu }$	${\delta }_{\theta }$	*l* = 2	*l* = 3	*l* = 4
0.0	0.0000	**358.352**	**367.099**	**365.266**
0.0	0.0025	298.243	330.178	316.783
0.0	0.0050	267.723	329.691	312.863
0.0	0.0075	243.771	328.451	307.073
0.0	0.0100	222.183	325.922	305.457
0.0	0.0125	202.478	323.687	302.749
0.0	0.0150	184.965	320.241	301.451
0.0	0.0175	168.504	319.265	300.062
0.0	0.0200	155.008	317.414	299.750
0.0	0.0225	142.375	316.542	299.181
0.0	0.0250	131.120	315.632	298.602
0.0	0.0275	121.341	314.440	297.293
0.0	0.0300	112.716	312.678	295.819
0.0	0.0325	105.268	310.584	294.309
0.0	0.0350	98.667	308.849	293.535
0.0	0.0375	95.652	307.863	290.535

ARL_0_ is in bold.

**Table 17:** ARLs for $\theta_{1} ,\theta_{2} = 0.1{\text{ and }}\theta_{3} = 0.8$ with shift in the attribute characteristics for *p* = 5.

Shift		Number of components *l*		
${\delta }_{\mu }$	${\delta }_{\theta }$	*l* = 2	*l* = 3	*l* = 4
0.0	0.0000	**379.101**	**355.901**	**385.003**
0.0	0.0025	322.233	307.696	334.383
0.0	0.0050	280.827	277.799	294.577
0.0	0.0075	248.247	247.568	263.277
0.0	0.0100	223.832	219.639	236.282
0.0	0.0125	203.811	196.142	215.775
0.0	0.0150	188.155	176.143	198.432
0.0	0.0175	174.738	159.336	184.118
0.0	0.0200	163.323	144.998	171.201
0.0	0.0225	153.144	132.619	159.992
0.0	0.0250	143.734	122.180	149.929
0.0	0.0275	134.248	113.207	140.121
0.0	0.0300	125.618	105.555	130.995
0.0	0.0325	117.642	98.954	122.644
0.0	0.0350	110.661	93.246	115.302
0.0	0.0375	107.574	90.770	112.057

ARL_0_ is in bold.

**Table 18:** ARLs for $\theta_{1} ,\theta_{2} = 0.05{\text{ and }}\theta_{3} = 0.9$ with shift in the attribute characteristics for *p* = 5.

Shift		Number of components *l*		
${\delta }_{\mu }$	${\delta }_{\theta }$	*l* = 2	*l* = 3	*l* = 4
0.0	0.0000	**351.264**	**367.627**	**357.014**
0.0	0.0025	316.049	314.919	316.022
0.0	0.0050	289.242	267.363	274.476
0.0	0.0075	267.386	230.531	235.027
0.0	0.0100	247.725	199.182	202.009
0.0	0.0125	230.936	174.150	174.736
0.0	0.0150	213.964	153.796	153.185
0.0	0.0175	196.540	137.891	135.997
0.0	0.0200	179.120	124.724	122.356
0.0	0.0225	163.074	113.962	111.383
0.0	0.0250	149.496	105.077	102.654
0.0	0.0275	138.568	97.935	95.760
0.0	0.0300	129.932	92.560	90.500
0.0	0.0325	123.585	89.106	87.195
0.0	0.0350	118.012	86.412	73.214
0.0	0.0375	109.982	81.422	70.323

ARL_0_ is in bold.

### Shift in variable and attribute characteristics

This subsection presents the performance of the proposed chart for detecting the shift in both variable and attribute characteristics. Table 19 reports the performance of the proposed chart for the balanced situation of attribute characteristics. Meanwhile, the proposed chart’s imbalanced and extreme performances are presented in Tables 20 and 21. From the results, it can be seen that there is a similarity performance with the performance of the proposed chart when it is used to monitor shifts in variable characteristics. The main difference in the performance lies in the type of shift. For small shifts, the proposed chart better monitors the shift in only variable characteristics. On the other hand, the shift in both variable and attribute characteristics produces better performance for the large shift.

**Table 19:** ARLs for $\theta_{1} ,\theta_{2} = 0.3{\text{ and }}\theta_{3} = 0.4$ with shift in the variable and attribute characteristics for *p* = 5.

Shift		Number of components *l*		
${\delta }_{\mu }$	${\delta }_{\theta }$	*l* = 2	*l* = 3	*l* = 4
0.0	0.0000	**383.134**	**358.421**	**355.567**
0.1	0.0025	333.432	327.743	340.012
0.2	0.0050	197.665	254.887	268.843
0.3	0.0075	19.332	265.425	252.876
0.4	0.0100	7.265	311.954	270.834
0.5	0.0125	5.123	176.021	186.598
0.6	0.0150	3.812	102.912	130.143
0.7	0.0175	3.032	56.765	85.722
0.8	0.0200	2.423	34.132	51.918
0.9	0.0225	1.976	19.932	34.764
1.0	0.0250	1.723	13.621	22.823
1.1	0.0275	1.551	8.754	14.921
1.2	0.0300	1.332	6.523	10.543
1.3	0.0325	1.281	4.821	7.222
1.4	0.0350	1.221	3.525	5.616
1.5	0.0375	1.108	2.732	4.023

ARL_0_ is in bold.

**Table 20:** ARLs for $\theta_{1} ,\theta_{2} = 0.1{\text{ and }}\theta_{3} = 0.8$ with shift in the variable and attribute characteristics for *p* = 5.

Shift		Number of components *l*		
${\delta }_{\mu }$	${\delta }_{\theta }$	*l* = 2	*l* = 3	*l* = 4
0.0	0.0000	**372.823**	**378.301**	**386.102**
0.1	0.0025	295.321	305.111	336.821
0.2	0.0050	448.865	214.923	257.601
0.3	0.0075	415.943	153.833	176.903
0.4	0.0100	303.663	109.232	137.789
0.5	0.0125	284.134	46.152	51.411
0.6	0.0150	149.639	31.512	32.443
0.7	0.0175	84.143	24.652	24.001
0.8	0.0200	48.022	19.212	17.862
0.9	0.0225	28.747	15.276	14.561
1.0	0.0250	17.702	12.901	11.809
1.1	0.0275	13.623	11.001	10.411
1.2	0.0300	10.511	9.381	9.003
1.3	0.0325	8.732	8.752	8.552
1.4	0.0350	7.111	7.431	7.863
1.5	0.0375	6.254	7.405	7.511

ARL_0_ is in bold.

**Table 21:** ARLs for $\theta_{1} ,\theta_{2} = 0.05{\text{ and }}\theta_{3} = 0.9$ with shift in the variable and attribute characteristics for *p* = 5.

Shift		Number of components *l*		
${\delta }_{\mu }$	${\delta }_{\theta }$	*l* = 2	*l* = 3	*l* = 4
0.0	0.0000	**370.911**	**393.532**	**340.631**
0.1	0.0025	382.241	358.722	259.873
0.2	0.0050	662.698	368.763	192.301
0.3	0.0075	655.372	352.601	132.004
0.4	0.0100	472.923	232.110	76.291
0.5	0.0125	26.653	30.942	17.129
0.6	0.0150	16.473	20.122	15.531
0.7	0.0175	15.683	17.065	15.165
0.8	0.0200	16.524	16.587	16.188
0.9	0.0225	18.734	18.892	17.542
1.0	0.0250	20.512	19.401	19.731
1.1	0.0275	21.614	20.981	23.129
1.2	0.0300	25.432	26.042	24.391
1.3	0.0325	27.476	29.366	28.561
1.4	0.0350	35.332	33.712	31.232
1.5	0.0375	38.622	37.902	38.808

ARL_0_ is in bold.

### Different correlation

This subsection presents the performance of the proposed chart for several coefficient correlations. In evaluating the performance of the proposed chart, the variable characteristics are generated with four types of correlation such as 0.3, 0.5, 0.7, and 0.9 using the KDE control limit. For this case, the process is shifted for both variable and attribute characteristics. The number of variable characteristics *p* is 5, and the number of principal components used *l* is 4. Also, the proposed chart is evaluated for three types of attribute characteristics as declared in the previous section.

Table 22 shows the performance of the proposed chart for monitoring the balanced proportion of attribute characteristics ($\theta_{1} ,\theta_{2} = 0.3{\text{ and }}\theta_{3} = 0.4$) with several types of correlation. The proposed chart always produces the ARL_0_ at about 370 for all scenarios for the in-control condition. The proposed chart can detect a shift when the process is shifted by producing smaller ARL_1_. For this case, better performance has achieved when the proposed chart monitors the process with a smaller coefficient correlation.

**Table 22:** ARLs of the proposed chart with *p* = 5, *l* = 4, $\theta_{1} ,\theta_{2} = 0.3{\text{ and }}\theta_{3} = 0.4$ for various correlation.

Shift		Correlation			
${\delta }_{\mu }$	${\delta }_{\theta }$	0.3	0.5	0.7	0.9
0.0	0.0000	**360.07**	**361.91**	**377.20**	**373.40**
0.1	0.0025	328.28	348.72	350.84	383.05
0.2	0.0050	300.59	314.70	322.56	351.08
0.3	0.0075	227.19	268.31	294.49	302.91
0.4	0.0100	169.96	208.13	226.09	254.12
0.5	0.0125	115.68	163.75	181.71	221.67
0.6	0.0150	87.60	121.27	149.54	174.47
0.7	0.0175	63.27	86.31	111.42	134.68
0.8	0.0200	43.65	67.61	88.84	108.28
0.9	0.0225	33.10	52.03	65.18	86.98
1.0	0.0250	24.41	37.79	50.80	66.99
1.1	0.0275	16.58	27.99	41.80	53.38
1.2	0.0300	13.06	21.54	32.74	43.11
1.3	0.0325	10.51	16.65	24.91	34.81
1.4	0.0350	7.90	13.85	19.44	27.78
1.5	0.0375	6.03	10.82	16.14	22.49

ARL_0_ is in bold.

Tables 23 and 24 reports the proposed chart’s performance in monitoring the attribute characteristics’ imbalanced and extreme imbalanced proportion. According to the tables, it can be concluded that for the in-control condition, the proposed chart produces the appropriate ARL_0_ (around 370 for $\alpha = 0.00273$). Similar to the previous result, the smaller coefficient correlation produces better performance, as seen from the ARL_1_ value for each scenario. In addition, the proposed chart reaches its peak performance when it is used in monitoring data in a balanced proportion of attribute characteristics.

**Table 23:** ARLs of the proposed chart with *p* = 5, *l* = 4, $\theta_{1} ,\theta_{2} = 0.1{\text{ and }}\theta_{3} = 0.8$ for various correlation.

Shift		Correlation			
${\delta }_{\mu }$	${\delta }_{\theta }$	0.3	0.5	0.7	0.9
0.0	0.0000	**382.21**	**384.078**	**386.722**	**377.494**
0.1	0.0025	354.866	369.03	352.675	374.787
0.2	0.0050	283.811	312.287	317.906	312.183
0.3	0.0075	227.887	249.544	246.147	259.416
0.4	0.0100	159.007	174.907	186.692	220.279
0.5	0.0125	113.073	137.541	142.863	165.263
0.6	0.0150	79.518	98.184	107.701	131.106
0.7	0.0175	58.335	72.649	87.998	98.644
0.8	0.0200	39.841	52.422	62.673	70.698
0.9	0.0225	30.111	39.531	45.567	55.034
1.0	0.0250	22.612	31.289	36.512	42.744
1.1	0.0275	17.746	22.203	27.707	32.644
1.2	0.0300	13.988	18.041	20.595	26.079
1.3	0.0325	11.212	14.613	16.773	20.683
1.4	0.0350	9.044	11.744	14.069	16.457
1.5	0.0375	7.909	10.679	11.865	13.758

ARL_0_ is in bold.

**Table 24:** ARLs of the proposed chart with *p* = 5, *l* = 4, $\theta_{1} ,\theta_{2} = 0.05{\text{ and }}\theta_{3} = 0.9$ for various correlation.

Shift		Correlation			
${\delta }_{\mu }$	${\delta }_{\theta }$	0.3	0.5	0.7	0.9
0.0	0.0000	**357.967**	**383.537**	**370.886**	**377.704**
0.1	0.0025	243.622	270.000	275.799	289.455
0.2	0.0050	149.216	158.257	168.138	181.141
0.3	0.0075	81.986	93.388	102.324	104.422
0.4	0.0100	49.656	54.899	59.872	54.953
0.5	0.0125	30.627	32.935	39.414	35.261
0.6	0.0150	22.166	22.090	21.672	24.862
0.7	0.0175	18.668	19.888	22.112	20.365
0.8	0.0200	18.578	18.370	19.078	18.643
0.9	0.0225	18.380	19.296	19.053	18.469
1.0	0.0250	19.638	20.959	19.975	20.525
1.1	0.0275	22.159	21.430	22.065	22.382
1.2	0.0300	24.402	24.746	24.343	24.081
1.3	0.0325	28.004	27.539	29.145	26.703
1.4	0.0350	31.852	33.243	32.269	32.048
1.5	0.0375	37.362	37.036	41.362	40.535

ARL_0_ is in bold.

Based on the simulation results about the performance of the proposed chart in monitoring process shift, the following findings can be summarized as follows:

1. The proposed chart is suitable for monitoring processes with shifts in variable characteristics and attribute characteristics with balanced proportions.
2. The proposed chart is suitable when used on quality characteristics of variables with low correlation and attribute characteristics with balanced proportions.

## Application in the real cases

### Machine failure dataset

This paragraph describes how the proposed chart is applied to a real-world scenario. The proposed chart is used to monitor of the machine failure dataset (attached as Excel file). This dataset have been used in Ref.^4^. There are 8784 samples in this dataset, along with 16 variable characteristics and 4 attribute qualities, one of which is labeling the observations. In this study, 8 out of 16 variable characteristics and 2 out of 3 attribute characteristics are chosen based on their mean deviation from the mean of the in-control process. While the second attribute characteristic contains four categories with a balanced percentage, the first attribute characteristic has eight with such ratio.

Table 25 shows the performance of the proposed chart in monitoring the Machine Failure dataset. According to the table, it can be seen that the performance of the multivariate based on the PCA Mix surpasses the performance of the conventional *T*^2^ chart. However, the PCA Mix chart with the *F* Distribution control limit has slightly better performance (see the Hit rate). Fortunately, the proposed chart demonstrates better performance than the other charts in detecting the real out-of-control observation. Based on the results, it can be seen that the proposed chart has better performance in detecting out-of-control signals compared to the others. This happened because the two attribute characteristics, which have a balanced proportion, increase the proposed method’s accuracy level.

**Table 25:** Proposed chart performance in monitoring the machine failure dataset.

Control chart	Control limit	Hit rate	FP rate	FN rate
PCA Mix with *F* distribution control limit	14.171	**0.99249**	0.00068	0.74074
*T*^2^ chart with *F* distribution control limit	23.590	0.99192	0.00126	0.81481
Proposed chart	11.507	0.99237	0.00241	**0.56790**

Significant values are in bold.

### NSL-KDD dataset

The well-known NSL-KDD dataset (available in https://www.kaggle.com/datasets/hassan06/nslkdd) is being monitored using the proposed chart in this section. It is regarded as a typical benchmark for assessing intrusion detection^38^. Table 26 details the proposed chart’s effectiveness in inspecting the NSL-KDD dataset. Based on the findings, we can see that the proposed chart performs better than the other charts. The proposed chart, which uses the KDE control limit, yields the highest hit rate and the lowest false positive rate.

**Table 26:** Proposed chart performance in monitoring the NSL-KDD dataset.

Control chart	Control limit	Hit rate	FP rate	FN rate
PCA Mix with *F* distribution control limit	14.1719	0.89707	0.03859	0.17662
*T*^2^ chart with *F* distribution control limit	23.5906	0.89151	0.09785	**0.12067**
Proposed chart	1.63280	**0.91136**	0.05688	0.12501

Significant values are in bold.

## Conclusions

This paper presents the detailed performance evaluation of the PCA Mix control chart in monitoring the mixed variable and attribute quality characteristics. Through some simulation studies with several cases, the performance evaluation shows the PCA Mix chart’s ability to detect outliers and shifts in the process. The proposed chart still has a stable performance for no more than 30 percent outlier mixed. When the proposed chart is used to monitor more than one attribute characteristic with a balanced proportion, most misdetection occurs due to false alarms for more than 30 percent of outlier. On the other hand, in monitoring the attribute characteristics with imbalanced proportion, the proposed chart cannot detect actual outliers when it detects more than 30 percent of outliers. Furthermore, the performance of the proposed chart is also evaluated in detecting a shift in the process. The proposed chart shows an outstanding performance in monitoring the shift in only variable characteristics for the small shift in the process. The proposed chart demonstrated better performance for the shift in both variable and attribute characteristics for the large shift in the process. The proposed chart has a better performance in monitoring the smaller coefficient correlation. In addition, the proposed chart is also applied to monitor two datasets, and its performance is compared with the conventional method. The monitoring results show that compared to the other charts, the proposed chart has a higher accuracy detection by detecting more actual out-of-control observations with a low false alarm rate.

For future research, the performance of the proposed chart can be extended by adding some robust estimator in both the mean vector and covariance matrix. The bootstrap resampling method can be used to estimate the control limit of the proposed chart. The Squared Prediction Error (SPE) or *Q* statistic can be employed as an alternative for Hotelling’s *T*^2^ statistic in monitoring the mixed characteristics. Also, the effect of autocorrelation for the metric data is interesting issue need to be explored.

### Ethics approval

This work does not involve experiments on animals and humans.

### Supplementary Information

Supplementary Information.

## Data Availability

The dataset is attached as a supplementary file.
